# Models of Care and Team Activities in the Delivery of Transgender Primary Care: An Ontario Case Study

**DOI:** 10.1089/trgh.2019.0082

**Published:** 2020-06-08

**Authors:** Erin Ziegler, Ruta Valaitis, Cathy Risdon, Nancy Carter, Jennifer Yost

**Affiliations:** ^1^ Daphne Cockwell School of Nursing, Faculty of Community Services, Ryerson University, Toronto, Canada.; ^2^ School of Nursing, Faculty of Health Science, McMaster University, Hamilton, Canada.; ^3^ Department of Family Medicine, McMaster University, Hamilton, Canada.; ^4^ M. Louise Fitzpatrick School of Nursing, Villanova University, Villanova, Pennsylvania.

**Keywords:** delivery of health care, Ontario, primary health care, transgender persons

## Abstract

**Purpose:** Transgender individuals experience barriers accessing primary care. In Ontario, primary care is delivered through a variety of delivery models. Literature supports team delivery of primary care for transgender individuals, yet little is known about care delivery in Ontario and the role of primary care teams. We intend to explore how primary care for transgender individuals is delivered within the different primary care models in Ontario and the roles primary care team members enact in care delivery, barriers, enablers, and clinical competence of practitioners in delivering transgender care.

**Methods:** Case study methodology was used to compare transgender care across three Ontario primary care models. Key informants identified cases known to provide transgender care for case selection. Qualitative interviews were conducted. Documentary evidence and field notes were collected.

**Results:** Practitioners clearly articulated their role and activities they were responsible for in providing care, however, they tended to work independently. In cases with an interdisciplinary team there was limited collaboration. Nurse practitioners, physicians, and counselors contributed most to the delivery of care. Key challenges included lack of service coordination within organizations, and the need for practitioner education. Continuing educational sessions, guidelines, and mentorship aided capacity building.

**Conclusions:** Providing primary care to transgender individuals is within the scope of practice for primary care practitioners and can be part of routine care delivered in different models of care. Primary care team collaboration can be strengthened by regular team meetings. Professional training needs to include transgender education and continuing education opportunities need development.

## Background

Primary care needs of transgender and nontransgender individuals are similar, however transgender individuals have unique needs related to medically supervised transition, including access to hormone therapy and transition-related surgeries.^1–3^ Transgender individuals continue to experience barriers accessing and obtaining care.^4^ Barriers include marginalization,^5,6^ access to a practitioner knowledgeable about transgender health care needs,^1,3,7–9^ lack of gender neutral washrooms,^10–12^ binary gender documentation,^9,12^ and inappropriate laboratory reference ranges.^10^ Furthermore, practitioners receive minimal education regarding transgender issues.^5,9^

There are ∼25 million transgender individuals worldwide,^13^ with ∼200,000 adults in Canada and 77,000 in Ontario.^14^ In Ontario, transgender health care is provided in primary care settings, however, there continues to be few practitioners providing transgender health services and long waiting times.^15^ Primary care in Ontario is delivered using multiple models of care delivery, including solo practitioners and interdisciplinary teams^16^ presenting a unique opportunity for study based on the different team make up, structures, and reimbursement in each setting. The model of care concept includes the organization and delivery of health care services.^17^ Ontario models of care are categorized based on payment and physician remuneration and include: Fee-for-Service (FFS); Family Health Networks; Community Health Centers (CHC); and Family Health Organizations.^16,18,19^ By 2012, 75% of Ontario's population and 75% of primary care physicians were enrolled in these models of care.^20^

FFS physicians practicing in small groups or as solo practitioners receive remuneration for each service as determined by the schedule of benefits.^16,21^ It does not include interdisciplinary teams and patients are not rostered. In the Family Health Networks and Family Health Organizations, physicians work in groups of three or more with a blended capitation salary, and patients are rostered.^22–24^ The blended capitation model requires contractual accountability to the Ministry of Health. Family Health Teams (FHTs) are colocated within Family Health Organizations where patients are rostered, and pay is based on blended-capitation or blended-salary payment.^16,21^ FHTs receive additional funding for interdisciplinary teams.^16,18,23^ CHC have salaried physicians and interdisciplinary teams, and provide services to “hard-to-serve” communities within a catchment area or defined patient population.^25^

Interdisciplinary collaboration and care coordination are key for the provision of transgender primary care.^26^ Literature supports a team approach to transgender primary care.^26–30^ However, little is known about how teams provide care. Key disciplines include physicians, nurse practitioners (NPs), nurses, mental health professionals, and pharmacists.^27,29–31^ Mental health professionals can be vital to the provision of transgender primary care.^29,31^

Little is known about how primary care is delivered to this population and how delivery differs between solo practitioners and interdisciplinary teams in Ontario. The following research questions were explored: how is primary care for transgender individuals delivered within different primary care models in Ontario? and what activities do interdisciplinary team members engage in when delivering primary care to transgender individuals? For this study, the term role is used to describe an individual's profession or job title, activity relates to the work individuals perform in their role. Study results can support primary care practices in the delivery of services to transgender individuals and inform capacity building.

## Methods

An exploratory multiple case study design was used.^32^ Normalization Process Theory (NPT) was used as a framework for the development of the study design and analysis. NPT is a framework used to understand and explain the dynamic processes that occur during implementation of interventions in health care.^33^ For this study, the delivery of primary care services was explored, while an exploration of influences on implementation are reported elsewhere.^34^ Using purposeful sampling, three cases were selected: a solo practice FFS, CHC, and FHT. A key informant from Rainbow Health Ontario (RHO) and the first author, identified eligible organizations known to provide primary care services to transgender individuals. Organizational consent was obtained, and participants were recruited by the primary author. Participants were eligible if they were currently involved in delivering health care services to or had contact with transgender individuals in the practice. Participants included physicians, NPs, nurses, allied health professionals, and clinical support staff. Nineteen individuals completed the study.

Case study data collection requires multiple sources of evidence.^32^ Methods included semistructured interviews (*n*=19), documentary evidence, and investigator field notes. On-site interviews ran between 25 and 100 min, using a structured interview guide (Table 1). All interviews were recorded and transcribed verbatim. Documentary evidence (e.g., reports, proposals, evaluations) were requested from each case. Unfortunately, there was a lack of documentary evidence, consisting only of a program proposal and transgender-affirming intake forms. There was not enough documentary evidence to contribute to the understanding of how care was provided. All data were analyzed using qualitative content analysis^35^ and cross-case synthesis^32,36^ in NVivo 11. Triangulation was facilitated by collecting and comparing data from multiple sources,^37,38^ using three cases to maximize the range of data,^37,38^ and working with a multidisciplinary research team experienced in primary care and qualitative research to support analysis and interpretation of results.^37,38^

**Table 1.** Semistructured Individual Interview Guide

Questions for practitioners	Questions for clinical support staff
(1) Can you tell me what primary care for transgender individuals means to you?	(1) Can you tell me what primary care for transgender individuals means to you?
(2) Tell me about yourself, what is your role in the organization?	(2) Tell me about yourself, what is your role in the organization?
(3) Tell me about your practice with transgender patients?	(3) How would you describe the primary care for transgender patients in your organization?
(4) How would you describe the primary care for transgender patients in your organization?	(4) I would like to know how easy or difficult it has been to integrate primary care for transgender patients into the work that you do, and any ways that your work has changed as a result?
(5) How easy or difficult it has been to integrate primary care for transgender patients into the work that you do, and any ways that your work has changed as a result?	(5) Tell me about team members' activities in the delivery of primary care to transgender patients?
(6) Tell me about team members' activities in the delivery of primary care to transgender patients?	(6) Is there any other information you want to give me about the provision of primary care services for transgender patients at your organization?
(7) If you could create the best possible primary care service/program for transgender individuals in Ontario, what would it look like?	
(8) Is there any other information you want to give me about the provision of primary care services for transgender patients at your organization?	

Credibility was enhanced with the consistent use of a semistructured interview guide.^37^ A field journal for researcher reflexivity and ideas was maintained to support credibility and confirmability.^39^ Transferability was established through thick descriptions of cases.^39,40^ Ethics Board approval was obtained from McMaster University Hamilton Integrated Research Ethics Board (HIREB No. 07-332).

## Findings

Case 1 was a FFS organization with one physician and one clinical support staff, who have provided care to ∼140 transgender individuals for 2 years. Case 2 was a FHT that has provided care to ∼70 transgender patients over the last 7–10 years. Their interdisciplinary team (*N*=20), were all involved in providing care to transgender individuals. Case 3 was a CHC (*N*=45) providing care to ∼300 transgender individuals. Participants were unable to identify how long Case 3 had been providing care for transgender individuals, stating they always had “a role with the LGBT community, that they've always been there” (Physician 2 CHC). Table 2 highlights key characteristics of each case and the number and type of staff who participated. The term practitioner is used to describe any health care practitioner who provides direct care to patients, whereas the term counselor includes mental health workers, such as social workers and psychotherapists.

**Table 2.** Case Characteristics

	Length of time providing transgender primary care	No. of transgender patients (approximation)	No. of staff participating in the study	No. of staff in the organization	Team members participating in the study						
					Physician	NP	Nurse	Counselor	Program manager	Community support worker	Clinical support staff
Case 1: Fee-For-Service	2 Years	140	2	2	1						1
Case 2: Family Health Team	7–10 Years	70	6	20	1	1	1	1	1		1
Case 3: Community Health Center	Unsure	300	11	45	2	2	2	3		1	1

NP, nurse practitioner.

The mix of team members varied between cases. Cases 2 and 3 included nurses, NP's, and other allied health practitioners, which were absent in Case 1. All participants described their roles and activities in providing primary care to transgender individuals (Table 3).

**Table 3.** Roles and Activities of Team Members

	Physician	NP^a^	Nurse^a^	Counselor^a^	Clinical support staff	Other^a^
Administrative duties					✓	✓
Advocacy	✓			✓		✓
Assessments	✓	✓	✓	✓		
Care coordination				✓		✓
Chronic disease management	✓	✓				
Counseling		✓		✓		
Episodic care	✓	✓				
Health promotion			✓	✓		
Medication teaching and administration of injections (hormones, vaccinations)			✓			
Order, monitor, and follow-up on diagnostic testing	✓	✓				
Patient education/teaching		✓	✓	✓		
Prescribing medication	✓	✓				
Preventative care	✓	✓	✓			
referrals	✓	✓		✓		

Other includes individuals who identified as a program manager or community support worker.

^a^ Excluded Fee-for-Service case.

### Participant roles and activities

Participants in all cases were clearly able to articulate their role and activities working with this population. However, some were unable to describe others' contributions. In team-based cases, nurses accurately described other team members' activities more often than others. Physicians and NP's were most involved in delivering primary care services to transgender individuals. Their activities were similar, most commonly, including patient assessments, counseling, providing transgender hormone therapy, and referrals to specialists for surgery. In all cases, physicians and/or NP's self-identified as the primary practitioner for transgender patients. Nonclinical staff were least involved.

Participants in all cases viewed the delivery of primary care to transgender individuals as no different from care provided to others: “I provide primary care to anyone the same way” (NP 2 CHC). Additionally, they all acknowledged accepting referrals for new transgender patients, identifying them as a priority population.

Overall, primary care services included preventative and episodic care, hormone therapy and monitoring, referrals for transition-related surgeries, counseling, and chronic disease management. Hormone therapy and access to transition-related surgeries are priorities for individuals^1^ and this need was recognized in all cases.

In terms of the specifics around diagnosis of gender dysphoria and initiation of hormones or other needs that they had, we've made efforts to do that … it's mostly been provision of medical services with potential referral to surgeons or other practitioners, as necessary. (Physician FFS)

Transition psychosocial readiness assessments are completed by different team members, depending on the cases. The solo FFS physician does all assessments and recommendations for treatment, and initiates hormone therapy. However, in team-based cases, patients are seen by multiple team members, before hormone therapy is initiated.

Two processes for initiation of hormone therapy were identified. The first was embedded directly in the primary care organization (FHT and CHC). Patients could see their primary care practitioner for a variety of issues, including hormone therapy. Physicians and NPs in Cases 2 and 3 addressed all aspects of primary care for their patients. The second process provided rapid access to hormones for patients whose current practitioner felt unable to initiate therapy (CHC and FFS). The program's goal is to improve access to hormone therapy. “It's an easy way for them to come in and start or continue their hormone therapy treatments. We have patients coming from all over Ontario to see us” (Clinical Support Staff FFS). Cases 1 and 3 provided patients with rapid access, which included seeing individuals on a short-term basis to initiate hormones, followed by a return to their regular primary care practitioner.

### Barriers and enablers in the delivery of care

#### Interdisciplinary collaboration

Transgender patients often see multiple practitioners to address their health needs or to complete a secondary surgical assessment. Case 3 participants identified barriers to team collaboration. For example, in Case 3 there was a division between the primary care and rapid access clinic. An internal policy caused a separation in these programs, making it difficult for patients to move across programs. A physician stated, “We sort of work in silos. I can't get my patients to connect with the other provider that works here [in the rapid access clinic]” (Physician 1 CHC).

Despite some barriers, participants in Cases 2 and 3 acknowledged the value of collaboration and teamwork as enablers.

It's been really beneficial to have a team … a specialised team … and different disciplines within the team so that … you can provide the best care possible for the person, so we're not doing this in isolation. (Counsellor 1 CHC)

Enablers in team-based care were case conferences or team meetings which provided opportunities to discuss patient needs and develop care plans. “It's very collaborative amongst the team. A social worker, nurse, and the practitioner are all working together to optimize the care that the client is getting. […]. So, we have time set aside for case conferencing” (NP 1 CHC). Case conferences also allow for greater understanding of roles to enable team functioning.

#### Coordination of external services

Finding practitioners who were knowledgeable about transgender issues and provided safe services was challenging for Cases 2 and 3. “Anticipating where it's safe to get your blood work done, where it's safe to go for an x-ray or go for a mammogram” (Physician 2 CHC). Practitioners needed to be aware of other local health care services providing gender-affirming and safe care. Sending an individual to another service that could potentially discriminate against them was reported to be a concern, as it could risk increasing the health disparities faced by this population.

Another challenge was coordinating access to surgical assessments that required two surgical assessment letters: “often, I'll know the person, and then no one else does. So how do I get a second letter?” (NP 2 CHC). It was also challenging to coordinate patients' assessments by a second team member.

#### Maintaining competency

Lack of formal and continuing education was identified in all cases. “It's always evolving, so I'm always wanting to seek training, to seek more knowledge because I feel like it's such a rapid changing field” (Counselor FHT). Participants in Cases 2 and 3 received continuing education from their organization, however, wanted further opportunities. Another barrier was being unaware of where to obtain training. “I almost feel like I don't know what's out there … if there is anything out there” (registered nurse 2 CHC).

Practitioners in all cases identified resources, such as guidelines, transgender-specific training, and conferences, as main avenues for developing competence. RHO's training and biannual conference^41^ and Sherbourne Health Center's guidelines^2^ were identified in all cases as primary references and sources for continuing education. Case 3 noted mentorship within the team. “I would mentor all of those practitioners.” RHO's mentorship teleconference was identified in all cases: “Every Wednesday is a trans health connection mentorship program where we can call in as providers in the community and troubleshoot, ask very specific questions” (Counselor FHT). In Case 1, mentorship outside the organization was particularly important: “Providers can participate and ask questions … specific questions or general questions or reach out to the group for information or just hear what's going on in the community in general. So, it's [trans mentorship call] another really excellent resource” (Physician FSS).

## Discussion

Participants clearly understood their roles in providing care, however, they were not always able to describe the roles and activities of others. It is not surprising that NPs and physicians contributed the most activities in providing primary care to transgender individuals. Generalized primary care, including acute episodic visits and preventive care, is managed by physicians and NPs, with support from the primary care team. Although no longer a requirement before starting hormones, mental health services constitute another large portion of primary care services.^10,42^ Counselors provide support by advocating, educating, and assessing patients for treatment.^31,43^ Key nursing activities involved initial assessments, medication administration, teaching, and preventative care updates, including vaccinations. Most practitioners reported working independently. Care coordination was only mentioned by counselors and a community support worker; however, it is likely others did this but did not mention it. Clinical support staff was responsible for administrative duties. The importance of team member roles was clearly identified. While team-based settings allowed transgender individuals to see a variety of practitioners, it is important to ensure that practitioners are not acting as the gatekeepers to accessing medically necessary treatment. While it may be necessary for some individuals to see multiple practitioners before receiving this care, it is not needed by everyone. Each transgender individual needs to be assessed to determine what resources or supports are needed to avoid delays in treatment and practitioners being seen as barriers to care.^44^

While NPs work has been optimized, primary care nurses are underutilized and not working to full scope of practice.^45^ Effective primary care nursing is economical and improves access, patient satisfaction, continuity of care, and health outcomes.^46–48^ Key activities and competencies include health assessments, health care management, education, health promotion, and counseling.^49–51^ Optimizing nursing roles has the potential to improve primary care access to transgender individuals.

Team meetings were discussed by some participants in Cases 2 and 3. Opportunities for shared communication through team meetings is critical in supporting effective interdisciplinary collaboration,^52,53^ and can aid in coordination and integration of transgender care.^28^ Team collaboration can be enhanced through shared goals, formal quality processes, and engaging practitioners to feel part of the team.^54^

Despite the presence of interdisciplinary teams in Cases 2 and 3, there were collaboration barriers. For example, difficulty accessing services from team members was a barrier in Case 3. Organizational leaders need to ensure that structural and relational supports are in place to support teamwork.^55^

There was no evidence to describe the optimal interdisciplinary teams needed for the provision of primary care to this population in either the FHT or CHC. The FFS solo practitioner delivered care to many transgender patients without team support. This leaves us questioning the optimal mix of primary care staff to support this population.

Comprehensive care to transgender individuals can be delivered by an interdisciplinary team or solo practitioner. However, challenges exist in finding external resources and practitioners that are knowledgeable, safe, and transgender friendly, such as a diagnostic imaging staff or a specialist. Referrals to outside individuals, such as counselors, medical specialists, or surgeons may be required, but essentially transgender patients should receive routine care from their primary care practitioner. In Ontario, transgender individuals are often left on waiting lists or travel great distances to receive health care.^15^ Primary care practitioners can improve access by routinely offering transgender care.

Practitioners developed competence through mentorship, guidelines, and continuing education opportunities. Improved education is needed to expand practitioners' knowledge regarding transgender health needs, treatment, and management. Advocating for curriculum development to include transgender content can help address barriers patients experience in finding a knowledgeable practitioner. Additionally, continuing educational opportunities need to be available for ongoing capacity building. Including transgender-specific content in primary care conferences, workshops, and training may also help.

## Limitations

This study presents an analysis at only one point in time and provides a snapshot into the delivery of transgender primary care in models that exist in Ontario. Results may not be generalizable to all primary care settings, but because of the purposeful selection of cases, could be transferable to similar models of care. Cases that had experience in the provision of transgender care were selected, thereby limiting transferability of results to those with little or no experience. However, results can inform similar practices interested in improving care to the transgender population. Future studies involving sites with less experience and other models of care are needed.

## Conclusion

Primary care practitioners providing care to transgender individuals are working hard to improve access to care and eliminate barriers. Providing primary care services to transgender individuals is within the scope of practice for primary care practitioners and can be part of routine care. This study identifies areas to further improve care delivery. Results support the development of educational initiatives that include the delivery of care to transgender individuals. More research is needed to focus on collaboration in teams providing care for transgender individuals and impact on health outcomes and patient experiences. The study's findings point toward the need for further research in Canada. It is important to note that making small changes based on this study, such as improvements to education and team collaboration could have a positive impact on the delivery of primary care for transgender individuals.

## Abbreviations Used

CHC: Community Health Centers
FFS: Fee-for-Service
FHT: Family Health Team
NP: nurse practitioner
NPT: Normalization Process Theory
RHO: Rainbow Health Ontario
